# Functional characterization of a manganese superoxide dismutase from *Avicennia marina*: insights into its role in salt, hydrogen peroxide, and heavy metal tolerance

**DOI:** 10.1038/s41598-023-50851-5

**Published:** 2024-01-03

**Authors:** Hamid Abedi, Azar Shahpiri

**Affiliations:** Department of Biotechnology, College of Agriculture, Isfahan University of Technology, Isfahan, 84156-83111 Iran

**Keywords:** Biochemistry, Biotechnology, Molecular biology, Physiology, Plant sciences

## Abstract

*Avicennia marina* is a salt-tolerance plant with high antioxidant and antibacterial potential. In the present work, a gene encoding MnSOD from *Avicennia marina* (AmSOD2) was cloned in the expression vectors pET28a. The resulting constructs were transformed into *Escherichia coli* strains Rosetta (DE3). Following the induction with Isopropyl β-d-1-thiogalactopyranoside, the protein His-AmSOD2 was expressed but dominantly found in the insoluble fraction of strain R-AmSOD2. Due to detection of mitochondrial transit peptide in the amino acid sequence of AmSOD2, the transit peptide was removed and AmSOD2 without transit peptide (tAmSOD2) was expressed in *E. coli* and dominantly found in the soluble fraction. The enzyme His-tAmSOD2 exhibited a molecular mass of 116 kDa in native condition. Nevertheless, in reducing conditions the molecular mass is 28 kDa indicating the enzyme His-tAmSOD2 is a tetramer protein. As shown by ICP analysis there is one mole Mn^2+^ in each monomer. The Pure His-tAmSOD2 was highly active in vitro*,* however the activity was almost three-fold lower than His-AmSOD1. Whereas the high stability of the recombinant His-AmSOD1was previously shown after incubation in a broad range pH and high temperature, His-tAmSOD2 was stable up to 50 °C and pH 6 for 1 h. The gene expression analysis showed that the gene encoding AmSOD2 is expressed in root, shoot and leaves of *A. marina*. In addition, the results show that the expression in the leaves was enhanced after treatment of plant with NaCl, H_2_O_2_, Cd^2+^ and Ni^2+^ indicating the important role of MnSOD in the resistant mechanism of mangroves.

## Introduction

Plants experience different environmental stresses in their life cycles^1^. Oxidative stress can be regarded as a complicated chemical as well as physiological phenomenon accompanying almost all biotic and abiotic stresses in higher plants; it is developed due to overproduction as well as accumulation of reactive oxygen species (ROS)^2^. ROS includes both oxygen radicals such as superoxide (O^⋅^), hydroxyl (OH^⋅^), hydroperoxyl (OH_2_^⋅^) and peroxyl (RO_2_^⋅^) radicals and molecules like hydrogen peroxide (H_2_O_2_), ozone (O_3_) and hydrochlorous acid (HOCL), which can be converted to radicals easily^3^.

ROS serves a significant function in numerous signaling pathways of the cells; however, the imbalance of ROS under such environmental stresses conditions as droughts, salinity, chilling, metal toxicity, UV-B radiation as well as pathogen can be extremely harmful to organisms, causing lipid peroxidation, protein oxidation, nucleic acid damage, inhibition of enzymes, and programmed cell death pathway activation^4,5^. Therefore, the cells are equipped with an endogenous antioxidant defense mechanism consisting of many non-enzymatic as well as enzymatic antioxidants, which can act both synergistically and interactively in neutralizing different free radicals^6,7^.

The non-enzymatic systems have been mostly mediated through low molecular mass antioxidants, like glutathione, ascorbic acid and flavonoids which can remove hydroxyl radicals as well as singlet oxygen^8^. Enzymatic systems include SOD, catalase, ascorbate peroxidase and glutathione peroxidase^9^.

The SOD is an important enzyme in protecting against the toxic impacts of oxidative stress through scavenging different superoxide radicals and boosting the related conversion into oxygen as well as hydrogen peroxide^10^. Four diverse classes of SODs can be considered, which depend on the metal which is present at the active center: manganese (Mn^2+^), iron (Fe^2+^), copper (Cu^2+^), or zinc (Zn^2+^). CuZnSODs are commonly found in cytosol related to eukaryotic cells as well as chloroplasts. MnSODs can be found in mitochondria, chloroplasts, and peroxisomes. Meanwhile, dimeric FeSODs, which are not present in animal cells, can be found in chloroplasts^11–13^. Salinity stress impacts in halophytes have been the subject of analysis by investigating the total protein activity of SOD and, through the transcript profiles of individual mRNAs which encode diverse SOD isoforms prior to and following salt stress treatments.

*Avicennia marina* is a group of mangroves that can grow in the tropics and subtropics of the world's estuaries^14^. Like other mangroves, *A. marina* can be regarded as compatible halophytes that survive in such severe environmental conditions as external salt concentrations and high light intensities commonly not suitable for the other plants' survival^15^. In *A.marina,* the total SOD activity was found to be enhanced in the leaf tissues following the six week period of applying salt stress^16^. Therefore, SOD seems to play an essential role in defense mechanism applied to respond to oxidative stress^17^.

Previously, we cloned and characterized a CuZnSOD (AmSOD1) from *A. marina*. The gene encoding this protein was heterologously expressed in *Escheriachia coli*^18^. The recombinant AmSOD1 showed high stability following incubation in a wide range of pH and high temperature which can be regarded as the most important feature of AmSOD1. In the present work, we characterized another SOD (AmSOD2) from *A. marina*. The obtained results displayed that this SOD could be a mitochondrial MnSOD. Following the transferring of gene encoding AmSOD2, this protein appeared mostly in the insoluble form in *E. coli*. However, due to the determination of mitochondrial target peptide at the N-terminal of AmSOD2, the heterologous expression of tAmSOD2 (AmSOD2 without target peptide) was also performed and it was dominantly found in the soluble form. The high production of this form of enzyme enabled us to purify and characterize the protein. In addition, the expression profile of AmSOD2 in the leaves of *Avicennia marina* was studied in the response to the various stresses.

## Results

### Sequence analysis

**Figure 1:**
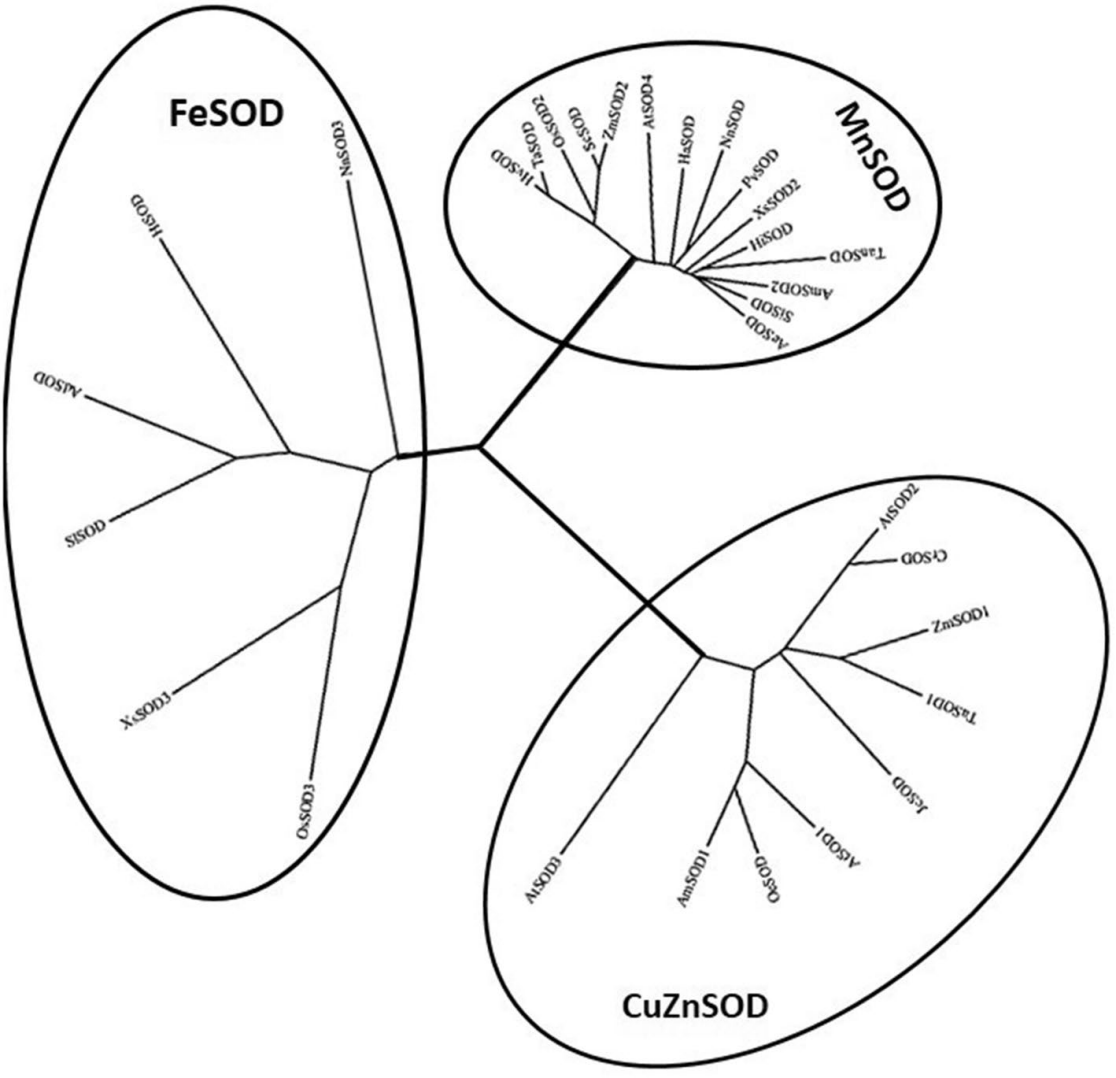
Phylogenetic tree of SOD sequences from different plants. For accession numbers and organisms, see “Materials and method”.

Multiple alignment including amino acid sequences of SOD (Supplementary Fig. S1, A) isoforms from different plant species was used to generate a phylogenetic tree (Fig. 1). The tree was divided into three major clusters. Cluster I contained the SODs characterized as CuZnSOD and their function was dependent on the presence of Cu^2+^ and Zn^2+^ as co-factors. MnSODs, characterized as plant mitochondrial SODs, belonged to Cluster II and FeSODs were classified in cluster 3. AmSOD1 and AmSOD2 were classified in clusters I (CuZnSODs) and II (MnSODs), respectively. The multiple alignments between the members of cluster II (Supplementary Fig. S1, B) showed that after amino acid 21, the sequences of the plant MnSODs were highly conserved during evolution (Supplementary Fig. S1, B), thus indicating that MnSODs may have a potential function in the stress tolerance mechanism in plants. The only region that seems to be very poor in conservation is between amino acids 4 and 21 (Supplementary Fig. S1, B).

### Gene expression profiling of AmSOD2

**Figure 2:**
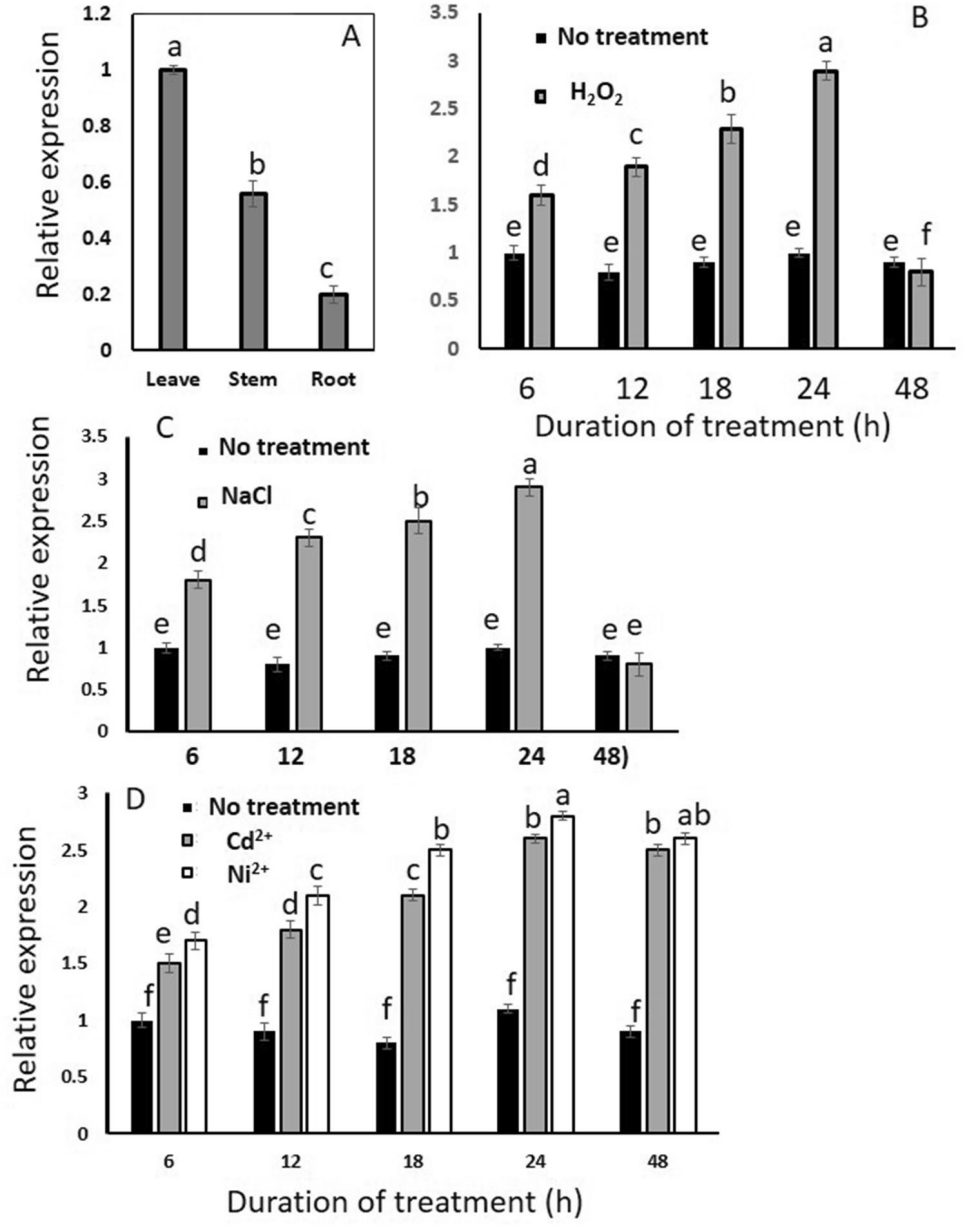
Gene expression of AmSOD2 in *Avicennia marina*. (**A**) Real-Time PCR analysis of gene encoding AmSOD2 in root, stem, and leaves of *Avicennia marina.* Real Time analysis of gene encoding AmSOD2 in the leaves in response to (**B**) H_2_O_2_, (**C**) NaCl, (**D**) CdCl_2_ and NiCl_2_ after different time points after treatment (6, 12, 18, 24 and 48 h) in the leaves. Expression level is shown as a value relative to that in control sample (plant with no metal treatments). Each histogram represents the mean ± standard deviation (SD) obtained from three independent biological replications. The statistical significance of the difference was determined by LSD test. Differences between treatments were considered significant when p < 0.05.

The expression of AmSOD2 using real time PCR showed that the gene encoding this enzyme was expressed in the root, stem, and leaves of *A. marina*. However, the expression in the leaves was significantly higher than that in the stem and root. Meanwhile, the expression in the root was much lower than that in the stem and leaves (Fig. 2A).

Whereas, in the plant leaves with no treatment, the expression level of the gene MnSOD was not changed over time, the expression of the gene in the leaves of H_2_O_2_-treated plants was significantly higher than that in the control plant. The expression in the treated plants was significantly enhanced during time up to 24 h. However, at 48 h, the expression of the gene in the leaves of H_2_O_2_-treated plants was similar to that of the control. These results, thus, showed that the expression of AmSOD2 was affected by H_2_O_2_ (Fig. 2B).

**Figure 3:**
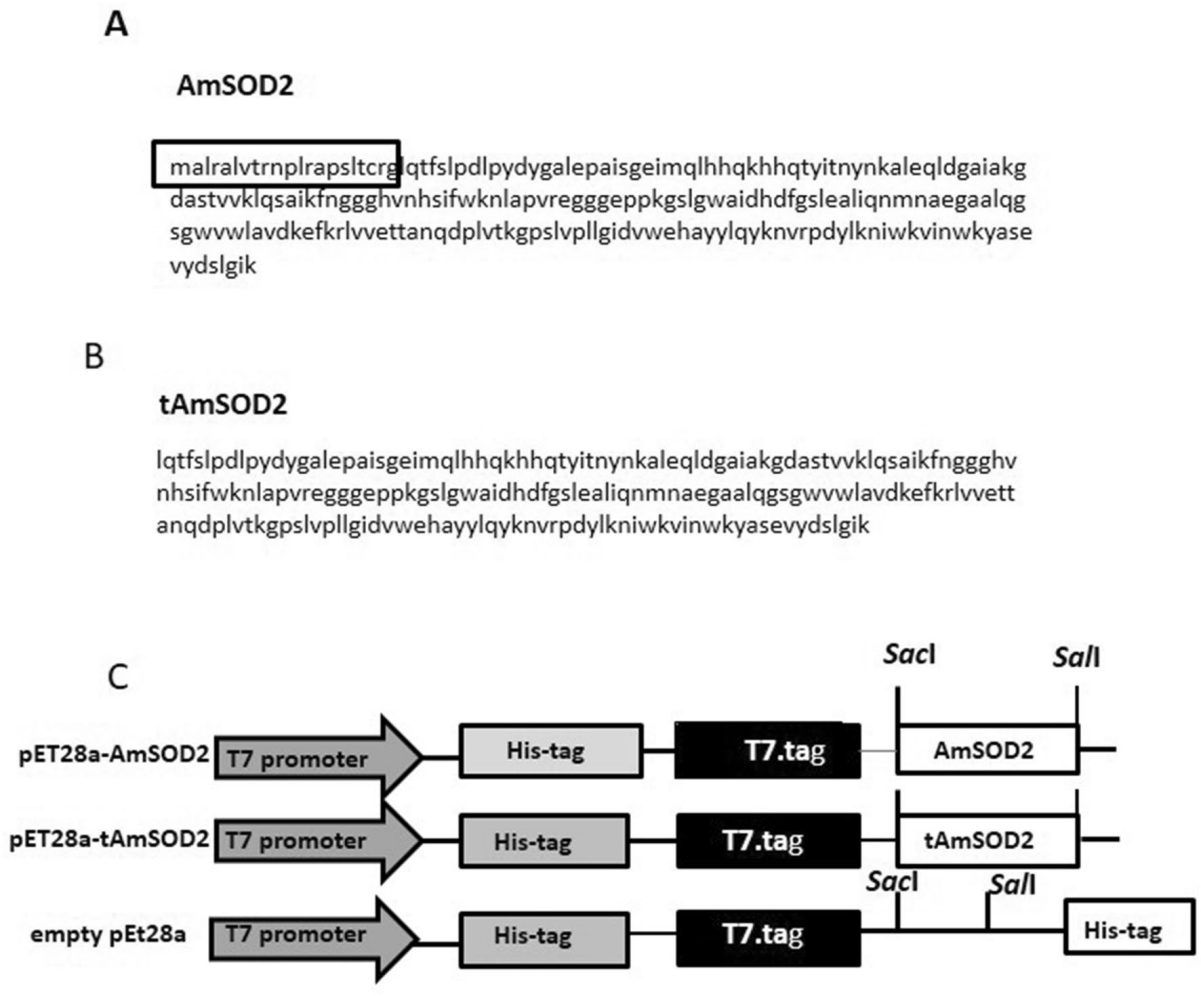
The sequence and map of construct. (**A**) The amino acid sequence of AmSOD2 and (**B**) tAmSOD2. (**C**) The map of pET28a-AmSOD2, The positions of promoter, His.tag and T7.tag and the gene are shown in the boxes.

The effect of NaCl on the expression of AmSOD2 was also evaluated in the leaves after treatment by NaCl. As shown in Fig. 3C, the expression of AmSOD2 was increased significantly up to 24 h and again decreased in the leaves following 48 h treatment (Fig. 3C).

In the plant leaves with no treatments, the expression level of AmSOD2 was almost similar during time; however, after treatment of plants with Ni^2+^ and Cd^2+^ was significantly increased up to 24 h and then remained constant (Fig. 3D). Therefore, the results showed that MnSOD is involved in the protection of plants in response to different stresses.

### Heterologous expression of AmSOD2, tAmSOD2 andAmSOD1

**Figure 4:**
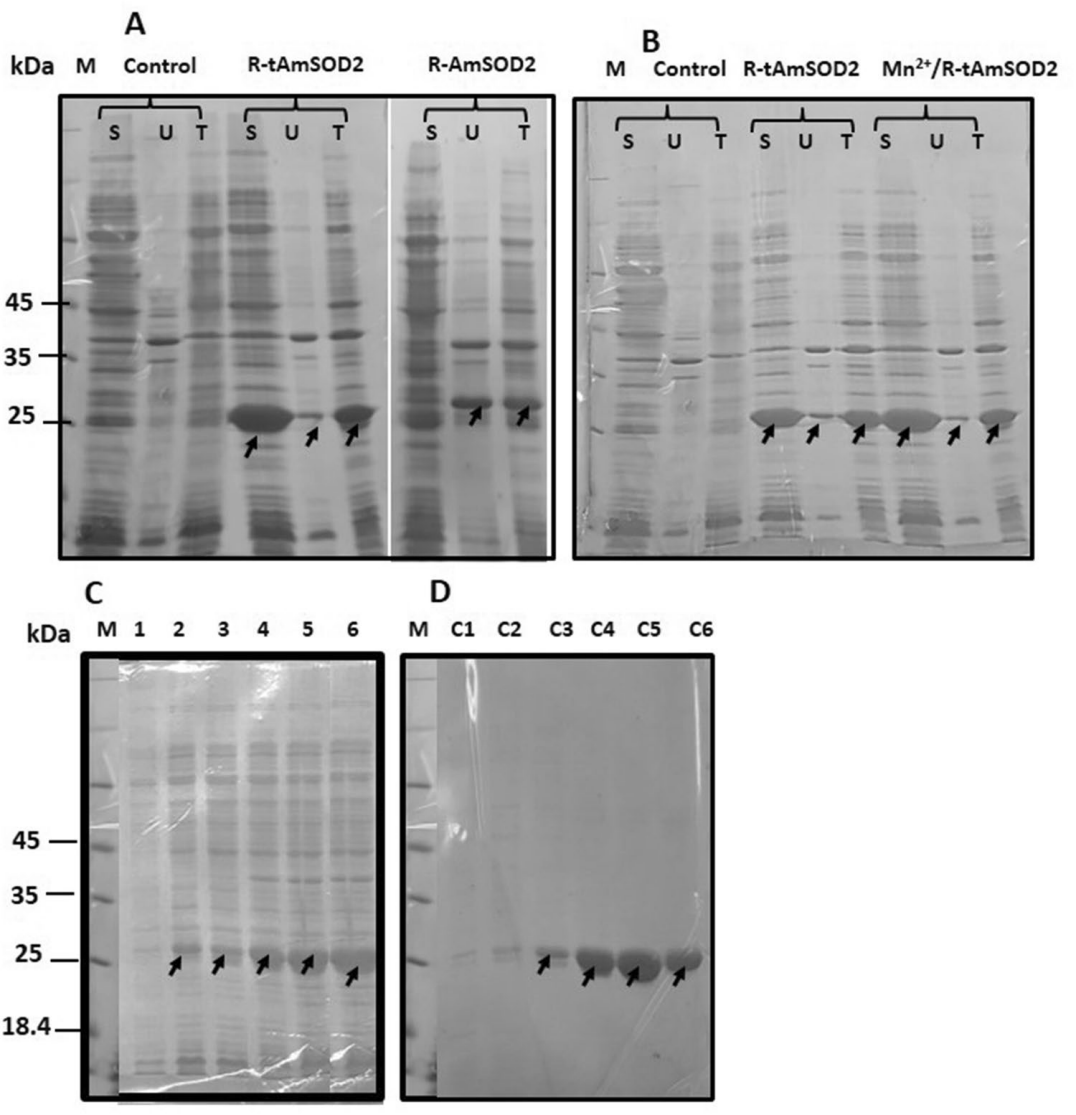
SDS-PAGE analysis and the solubility of expressed AmSOD2 and tAmSOD2. (**A**) Total soluble protein extracted from control strain, R-AmSOD2, and R-tAmSOD2 after 4 h induction with IPTG. The study of solubility of His-AmSOD2 and His-tAmSOD2 by comparison of band intensities in the total protein (T), the soluble fraction (S) and the unsoluble fraction (U) extracted from R-AmSOD2 and R-tAmSOD2, respectively (The recombinant proteins were shown with arrows). (**B**) The effect of supplementation of medium with Mn^2+^ on the solubility of His-tAmSOD2 (**C**) The enhancement of His-tAmSOD band during time up to 4 h after induction with IPTG (lanes 1–6; 0, 0.5, 1, 2, 3 and 4 h after addition of IPTG) (**D**) SDS-PAGE analysis for confirmation of quality of His-tAmSOD2 after purification with affinity chromatography. The fractions C1-C6 are the pure proteins that were eluted from His-tag columns by a gradient of 10–200 mM imidazole.

The gene which encodes AmSOD2 has 681 base pairs that can encode a protein which contains 226 amino acids (Fig. 3A). However, as predicted, a 21 amino acid sequence from N-terminal seems to be a mitochondrial signal peptide (Fig. 3B). As the gene was cloned in pET28a (Fig. 3C), the protein would be produced as a fusion one with an N-terminal His. Tag. The growth of the strain R-AmSOD2 (Rosetta stain carrying pET28a-AmSOD2, Fig. 3B) was done in the LB medium with kanamycin and chloramphenicol (Table 1). The Rosetta strain which contained empty pET28a was taken as the control strain (Table 1). After induction with IPTG, the expression of the recombinant protein His-AmSOD2 was analyzed in the total (insoluble fraction + soluble fraction) and insoluble fractions belonging to the R-AmSOD2 extract. The theoretical molecular weight obtained for His-AmSOD2 was found to be 29 kDa. SDS-PAGE of cell extracts displayed a remarkable polypeptide band of the expected molecular mass (Fig. 4A). Despite this, the band appeared weakly in the soluble fraction, which indicated that the protein had been mostly formed as the inclusion body. The corresponding band could not be seen in the considered control strain.

**Table 1:** The list of constructs and strains that has been provided in this research.

Strain	Expression host	Vector	Recombinant protein	Molecular weight	Research
R-AmSOD1	Rosetta (DE3)	pET28a-AmSOD1	His-AmSOD1 (CuZnSOD)	38	Fesharaki et al.^18^
R-AmSOD2	Rosetta (DE3)	pET28a-AmSOD2	His-AmSOD2 (MnSOD)	29	This research
R-tAmSOD2	Rosetta (DE3)	pET28a-tAmSOD2	His-tAmSOD2 (MnSOD)	26.8	This research
Control	Rosetta (DE3)	pET28a	His-tag	0.8	This research

As explained above due to the prediction of a mitochondrial target peptide, the expression of tAmSOD2 (AmSOD2 which does not have the target peptide) was investigated in the strain R-tAmSOD2 (Table 1). The theoretical molecular weight for His-tAmSOD2 was 26.8 kDa. As shown in Fig. 4A, the removal of target peptide caused the protein to be dominantly found in the soluble fraction, which could suggest that the target peptide presence might have interference with the protein folding in *E. coli* cells. In addition, as shown in Fig. 4B, the His-tAmSOD2 solubility was slightly enhanced by the supplementation of the medium by MnCl_2_ during expression.

The concentration of produced His-tAmSOD2 increased until 4 h following the IPTG addition for both His-AmSOD2 and His-tAmSOD2 (Fig. 4C). Therefore, for the large-scale production and purification, harvesting of the cells was done 4 h following induction by IPTG.

Purification of the recombinant His-tAmSOD2 from the soluble fraction was done by applying nickel affinity chromatography in the yields of 30 mg/L. The purification quality was evaluated through SDS-PAGE analysis (Fig. 4D, Fractions C3, C4, C5, and C6).

To compare the activity of His-tAmSOD2 with His-AmSOD1, the protein His-AmSOD1 was also produced and purified as previously explained^18^.

### The molecular mass of native His-tAmSOD2

**Figure 5:**
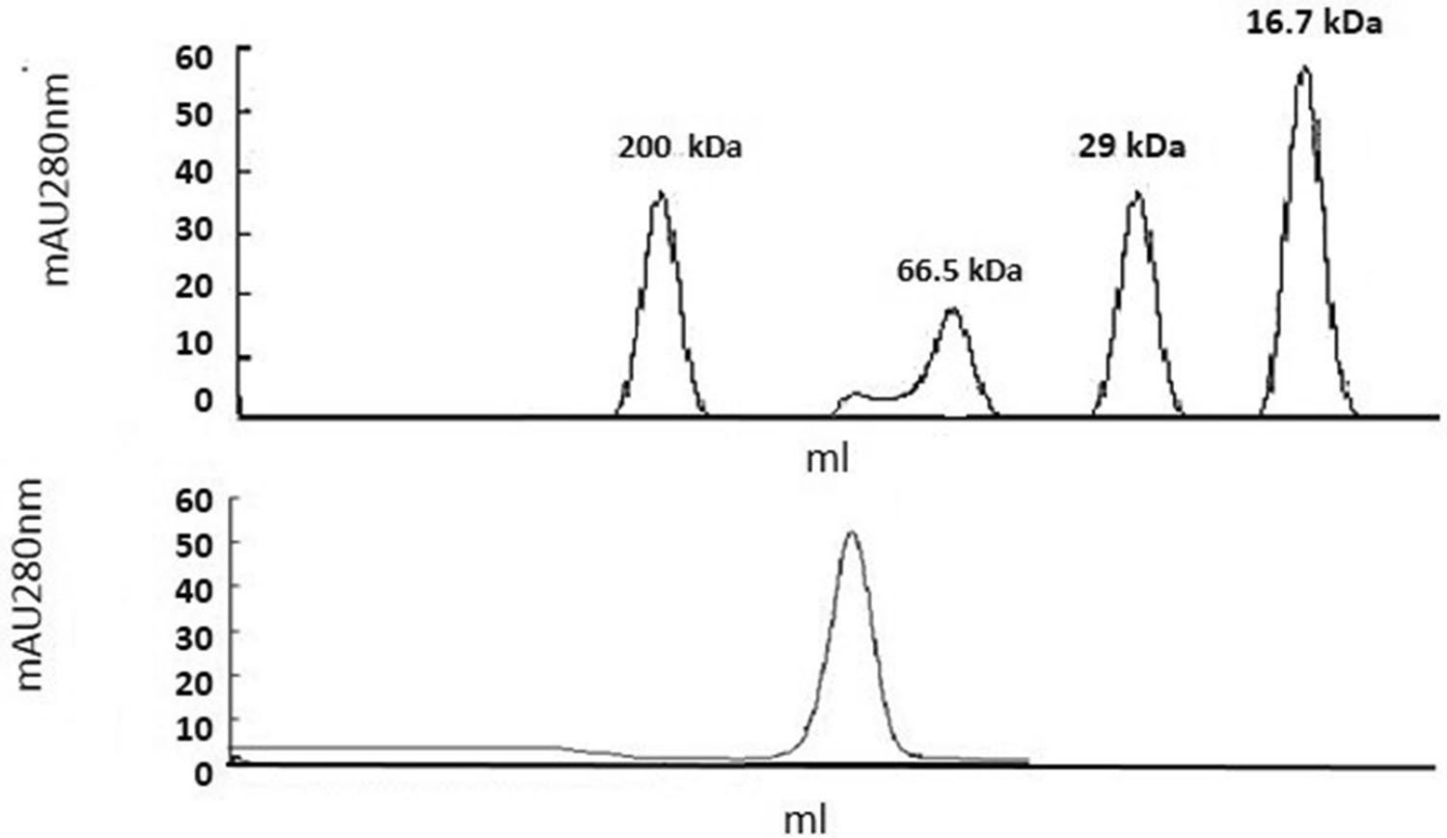
Molecular mass of purified His-tAmSOD2 using gel filtration. (**A**) Native molecular mass of recombinant His-tAmSOD2 was measured with respect to standard protein marker as resolved in Biosil-250 analytical gel filtration column. Standard proteins used were: (a) 200 kDa (b) 66.5 kDa-BSA fraction (c) 29 kDa-Carbonic anhydrase and (d) 16.7 kDa human myoglobin.

Here, based on gel filtration chromatography on a calibrated column, a solution molecular mass of almost 116 kDa was observed for His-tAmSOD2 before denaturation and reduction. However, due to the treatment of His-AmSOD2 by SDS and DTT, a single band in the position of 26 kDa was observed. These results, thus, suggested that His-tAmSOD2 could be a homotetramer (Fig. 5).

Correspondingly, according to the previously obtained results, prokaryotic MnSODs could be considered dimeric and eukaryotic MnSODs are, for the most part, tetrameric^19^. Previous studies have also revealed that soybean as well as Arabidopsis chloroplast CuZnSODs were tetramers, while peroxisomal and cytosolic CuZnSODs were taken as dimers^20^; MnSOD was a tetramer and FeSOD could be considered a monomer^20^.

### SOD activity

SOD activity was determined for the extracted soluble protein from R-AmSOD1, R-AmSOD2 and R-tAmSOD2, as well as the control strain (containing empty plasmid). As shown in Table 2, SOD activity for the extracted protein from the strains R-AmSOD2, R-tAmSOD2 and R-AmSOD1 was 7, 32 and 32-fold higher than that in the extracted protein from the control strain. The SOD activity in the extracted protein from R-tAmSOD2 was twofold higher than that from the strain R-AmSOD2, which suggests that the presence of transit peptide interferes with enzyme activity. When the culture medium for the strains R-AmSOD2 and R-tAmSOD2 was supplemented with Mn^2+^, the SOD activity was significantly increased, indicating the key role of Mn^2+^ in the function of His-AmSOD2 and His-tAmSOD2. Correspondingly, according to a previous study, the presence of Cu^2+^ and Zn^2+^ was also very efficient in the AmSOD1 activity^18^.

**Table 2:** SOD activity in the protein extract of strains R-AmSOD1 and R-AmSOD2.

Strain	Cu^2+^ (µM)	Zn^2+^ (µM)	Mn^2+^ (µM)	SOD activity (U/mg crude protein)
R-tAmSOD2	–	–	–	448 ± 8 b
R-tAmSOD2	–	–	250	479.2 ± 8 a
R-AmSOD2	–	–	–	107.9 ± 5.5 d
R-AmSOD2	–	–	250	236 ± 5.5 c
R-AmSOD1	250	250	–	475.5 ± 6 a
Control strain	–	–	–	14.8 ± 2 e

Data presented in this table are mean ± standard deviation (SD) from three independent biological replications. The statistical significance of the difference was determined by LSD test. Lowercase letters indicate significant differences between treatments at p ≤ 0.05.

The activity of purified His-tAmSOD1 and His-tAmSOD2 was also determined in the presence of Mn^2+^ and Cu^2+^/Zn^2+^, respectively (Table 3). In comparison to His-tAmSOD2, His-AmSOD1 showed fourfold higher activity.

**Table 3:** Activity of purified His-AmSOD1 and His-tAmSOD2.

Strain	Purified recombinant protein	Cu^2+^ (µM)	Zn^2+^ (µM)	Mn^2+^ (µM)	SOD activity (U/mg purified protein)
R-AmSOD2	His-tAmSOD2	–	–	250	1600 ± 25
R-AmSOD1	His-AmSOD1	250	250	–	5962 ± 10

### Metal content of His-tAmSOD2

The enzyme His-tAmSOD2 metal content, which was produced by R-tAmSOD2 in the medium which contained MnCl_2,_ was determined through ICP-MS. The obtained results showed that Mn^2+^ was 3.8 ± 0.1 Mn^2+^ mol per mole tetramer of the recombinant His-AmSOD2. Correspondingly, the plant MnSODs which have been characterized in other plants are tetramer, with four monomers, and each monomer contains an active site bound to a Mn^2+^ ion^21^.

### The effects of temperature and pH on the activity of tAmSOD2

**Figure 6:**
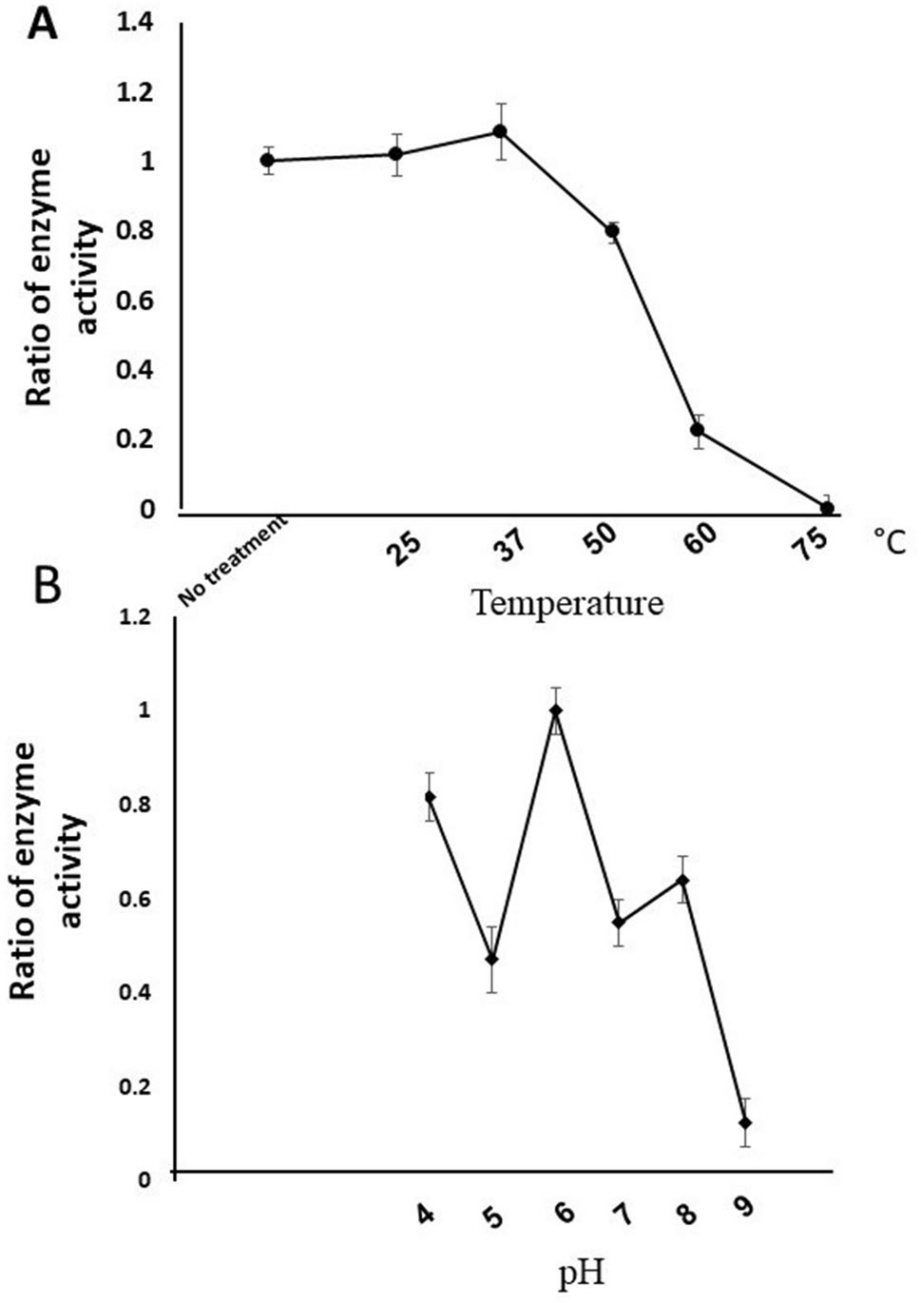
Effects of incubation at different temperatures and pH on the activity of His-tAmSOD2. The purified His-tAmSOD2 was incubated (**A**) at temperatures (25, 37, 50, 60 and 75 °C) (**B**) in buffers with pH (3–12) for 1 h. For temperature, the ratio of activities was calculated based on the activity of fresh pure recombinant His-tAmSOD (No treatment). For pH, the ratios were calculated based on the activity in pH 6. Each data point represents the mean ± SD obtained from three independent reactions.

The activity of His-tAmSOD2, produced in the medium containing Mn^2+^, was determined after incubating the enzyme at diverse temperatures for 1 h, as compared to a fresh purified His-tAmSOD2, which was not treated (Fig. 6A). The purified His-tAmSOD2 displayed 100% of its activity after incubation at 25 °C and 35 °C for 1 h. Even about 80% of SOD activity remained after incubation at 50 °C for 1 h. Nevertheless, at 60 °C, only 20% of the activity was kept and at 75 °C, the protein completely missed its activity.

Regarding to pH stability, the activity was at maximum amount when the protein was incubated at pH 6 for 1 h. In comparison to this amount, the results showed that His-tAmSOD2 maintained 47% of its activity after incubation at pH 4 for 1 h (Fig. 6B). The activity decreased to 50–60% at pH 7–8. However, only 12% activity remained for this enzyme after incubation at pH 9.

**Figure 7:**
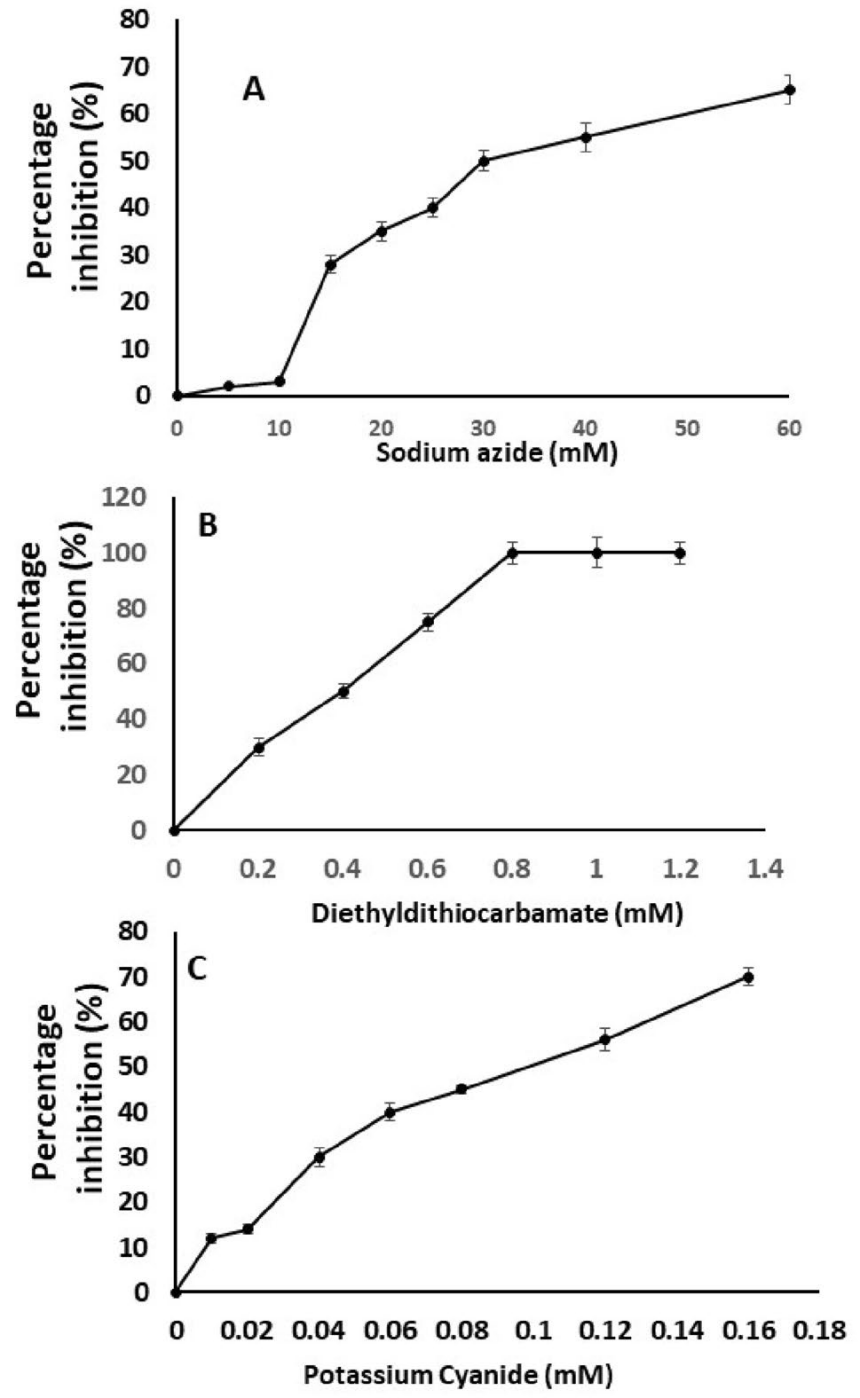
The effects of inhibitors on the activity of recombinant and pure His-tAmSOD2. (**A**) Sodium azide, (**B**) diethyldithiocarbamate, (**C**) potassium cyanide.

Results related to the effect of various inhibitors on the His-tAmSOD2 activity have been shown in Fig. 7; 50% of the native activity of tAmSOD2 was inactivated in the presence of 30 mM of sodium azide, 0.4 mM diethyldithiocarbamate and 0.12 mM potassium cyanide. Therefore, it seems that His-tAmSOD2 is the least sensitive to sodium azide and most sensitive to potassium cyanide. Nevertheless, generally, according to the previous studies, CuZnSODs are more sensitive than MnSODs to diethyldithiocarbamate and potassium cyanide because of the effects of these inhibitors on removing Cu^2+^ from enzyme^22^. In contrast, there are also reports showing that MnSODs are more sensitive than CuZnSODs^23^.

### Longevity of tAmSOD2

The protein His-tAmSOD2 is completely soluble and active in imidazole before dialysis. In this situation, it can be kept at 4 °C without any loss of activity for up to 3 months. However, storage at − 20 °C maintains the activity of His-tAmSOD for a longer time (up to 6 months).

Dialysis of protein against potassium phosphate buffer resulted in the precipitation of almost 25% of protein, which could be removed by filtration. After filtration, the rest of protein in potassium phosphate buffer could not be stored at + 4 °C since more protein started to precipitate. However, storage of protein at − 20 °C caused the protein to remain active up to 3 months without precipitation.

## Discussion

The mangrove *A. marina* as a salt-tolerant plant, confers salt-mechanism tolerance including osmolyte accumulation, efficient salt secretion through salt glands and antioxidant system^24–26^. Roots are known to be in direct contact with the outside environment, playing the first guard in avoiding the phytotoxins’ harm to plants. Salt exclusion in the root seems to act as the first barrier in withstanding high salinity in mangroves^27^. Despite this, the generation of ROS can be regarded as a main representation of salt stress^28^. To tackle such a challenge, mangroves apply such strong antioxidant systems as SOD, catalase and peroxidases which could be very efficient in highly saline microenvironments^29^.

Although transcriptome analysis in *A. marina* has yielded discrepant results concerning the role of AmSODs in the tolerance of *A. marina* in response to salt and other oxidative stresses^28^*,* encoding AmSOD1 confers tolerance to, salinity, and drought stress, as compared to untransformed control plants^30^.

The heterologous expression of the full length of His-AmSOD2 in *E. coli* was not successful because the recombinant protein was produced as the insoluble form, which suggested that the protein was not folded properly. However, the heterologous expression of tAmSOD2 (the protein without predicted mitochondrial transit peptide) resulted in finding the recombinant protein dominantly in the soluble fraction. These results, in addition to other properties, such as detection of a mitochondrial transit peptide in amino acid sequence, the tetrameric native of AmSOD2 and also, determination of four Mn^2+^ ions per each mole of tAmSOD2, thus confirmed that AmSOD2 could be regarded as a mitochondrial SOD. Based on the previous studies, it is very well documented that plant MnSODs are particularly important antioxidant enzymes since they are located in mitochondria, representing the first defense line against superoxide radicals which are produced as the byproducts of oxidative phosphorylation^31^; also, it is assumed to be a major scavenger of damaging ROS in the mitochondrial matrix^32^. The mitochondrial proteins synthesized in cytoplasm could not be imported into mitochondria in their very native state^21^. Consequently, proteins are unfolded during the translocation process normally and threaded through the import machinery as linear chains^6,33^. Correspondingly, the mitochondrial target peptide was also previously detected in the sequence of different plant MnSODs such as *Triticum acetivum*^34^ and *Saccharum officinarum*^35^.

The SOD activity was significantly enhanced by removing target peptide. However, the activity of His-AmSOD1 is four times higher than that of His-tAmSOD2. In addition, similar to His-AmSOD1, which had higher activity with both Zn^2+^ and Cu^2+^, the presence of Mn^2+^ was very effective on both solubility and activity of tAmSOD2. The activity of pure recombinant His-AmSOD1 (6000 U/mg) was even higher than that of bovine CuZnSOD (2500–3000 U/mg)^36^. However, the activity of His-tAmSOD2 with 1600 U/mg was less than that of human MnSOD (almost 5000 U/mg) which had been purified from the liver by a three-step method^37^.

Whereas His-tAmSOD2 is a thermostable enzyme and 80% of its activity is kept after incubation at 50 °C for 1 h, the enzyme is completely inactivated after incubation at 75 °C. In comparison, 50% of AmSOD1 activity remained even at 75 °C^18^. Overall, almost all reported SODs were inactivated at the temperature of 70 °C, except for AmSOD1 and CuZnSODs from *A. camphorate* (AcSOD) and *Hyperthermophilic archaeon (HaSOD), Sulfolobus acidocaldarius* (ScSOD), *Aeropyrum pernix* (ApSOD) and *S. solfataricus*^38–41^.

As shown in *A. marina* and some other plants, the thermostability of CuZnSOD is higher than that of MnSOD; however, in fungi, the MnSOD from *T. aurantiacus* var. levisporus is more than Cu/ZnSODs of *Aspergillus flavus*, *A. niger*, *A. nidulans*, *A. terreus*^42^, and *A. fumigatus*^43^.

*The* gene encoding AmSOD2 is expressed in different organs of *A.marina,* including the root, stem and leaves, before induction by stress. The highest expression was in the leaves, while the lowest one was in the root. Previously, Corpas et al. had shown that different SOD isozymes including CuZnSOD, MnSOD and FeSOD were expressed in the leaves; however, they were differentially expressed in different cell types of leaves^44^. Here we also showed that the expression of AmSOD2 was significantly enhanced in the leaves by treating *A. marina* by H_2_O_2_, NaCl and heavy metals Ni^2+^ and Cd^2+^, thus suggesting the involvement of AmSOD2 in the stress tolerance in mangroves. Correspondingly, previous studies have shown that SODs are influenced in response to stresses leading to oxidative stress. The previous studies have also shown the enhanced expression of ScMnSOD in response to the fungal pathogen *Ustilgao scitaminea* in sugarcane^35^**,** up-regulation of SaSOD1, a CuZnSOD from *Saccharum arundinaceus*, in response to drought^45^, and up-regulation of TaSOD, a CuZnSOD from *Triticum aestivum,* in response to heat as some of these examples.

In addition, the transgenic rice, tobacco, sweet potato and Arabidopsis, which heterologously express a member of plant CuZnSOD family, showed resistance to salt and drought^46–49^. Transfering of tobacco *MnSOD* gene into *Medicago truncatula* led to improving the cold-resistance ability, thus raising the yield output^50^. Transgenic *Brassica* plants over-expressed wheat *MnSOD* gene, displaying more tolerance to oxidative stress as well as aluminum toxicity^51^.

## Conclusion

The gene encoding AmSOD2 is expressed in the root, stem and leaves in 1-month-old *A. marina* seedlings. In response to NaCl, H_2_O_2_, Ni^2+^ and Cd^2+^ the expression of this gene was significantly enhanced in the leaves. Due to the presence of mitochondrial transit peptide in amino acid sequence of AmSOD2, this protein without the N-terminal transit peptide (tAmSOD2) was heterologously expressed in the soluble fraction of *E. coli*. In addition, the characterization of recombinant form of tAmSOD2 confirms that tAmSOD2 is a tetramer protein with four Mn^2+^ per mole. These properties declare that AmSOD2 is a MnSOD that is found in the mitochondrion. The protein is stable after incubation of protein up to 50 °C or pH 6 for 1 h.

## Materials and method

### Plant materials

Seeds of *A. marina* were collected from the mangrove forest, Gheshm, Iran. They were grown in sand-filled trays in a greenhouse at 35 ± 2 °C under a 16 h/8 h light/dark photoperiod and irrigated once time every day with tap water for 1 month^18^.

### Preparation of the samples for gene expression analysis

To conduct the gene expression analysis of gene encoding AmSOD2 in different organs the leaves, stems, and roots of 1-month-old *A. marina* seedlings (four-leaf stage) were harvested and stored at the temperature of − 80 °C until use.

Regarding the gene expression analysis of AmSOD2 under salt, hydrogen peroxide and heavy metals, 1-month-old *A. marina* seedlings (four-leaf stage) were acclimatized for a period of 3 days under 16 h light/8 h dark cycle in a growth chamber with half-strength MS medium^52^. The pre-adapted plants were then transferred to a half-strength MS medium supplemented with 0.5 M NaCl (near the NaCl concentration in the sea^53,54^. Leaf tissues were harvested from the time intervals of 0, 12, 18, 24 and 48 h of the salt stress treatment. For hydrogen peroxide, Ni^2+^ and Cd^2+^ treatment, the seedlings were transferred to a half-strength MS medium supplemented with 90 mM H_2_O_2_, 250 mg L^−1^ CdCl_2_ or NiCl_2_, respectively. Then the leaves were harvested at the time intervals of 0, 12, 18, 24 and 481 h after stress treatment and stored at the temperature of − 80 °C until use.

### Real time PCR

Extraction of total RNA from the samples of the plant material was done by applying the High Pure RNA isolation Kit (Roche) and then treated with RNase-Free DNase (Thermo Scientific) for the removal of the genomic DNA contamination. Total RNA (0.1 µg) was then reverse transcribed (RT) for the synthesis of the first strand cDNA by applying AMV reverse transcriptase (Thermo Scientific) and oligo dT primer (Thermo Scientific) based on the manufacturer’s recommendations. For the PCR reaction, the designing of specific primers was done using software Invitrogen Oligo Perfect TM Designer (http://tools.invitrogen.com/content.cfm). The sequences of forward and reverse primers were 5′GATCATGCAACTCCACCACC3′ and 5′ TCACGGACAGGAGCAAGATT3′, respectively. Real-time PCR was then done with a total volume of 20 µl that contained 10 µl Real Q Plus 2× master mix, 1 µl (0.5 µM) from each primer, 2 µl cDNA (1:15 dilution of the prepared cDNA) as the template, and 6 µl RNase free water. The related reactions were carried out with an initial 15 min denaturation at the temperature of 95 °C and this was followed by 40 cycles of 95 °C for 30 S, 57 °C for 30 S, and 72 °C for 30 S; this was then followed by a final extension at the temperature of 72 °C for a period of 5 min. Calculation of normalized expression levels was done by employing the ΔΔCt method^55^. With *Avicennia marina* actin (HaACT) as the reference gene. The calculation was on the basis of two technical and three biological replicates.

### Plasmid construction and preparation of strains

The gene which encoded AmSOD2 (NCBI Accession Number: AY137205.1) was codon- optimized, synthesized (Gene Universal) and cloned in pUC57 between two restriction sites *Sac*I and *Sal*I*.* To sub-clone gene in the pET28a expression vector, the digestion of plasmid pUC57-was done with *Sac*I and *Sal*I and the product of digestion was run on the agarose gel. Then isolation of the fragment which contained the AmSOD2 gene from the gel was done. The ligation of the fragment into pET28a was done, as the expression vector following linearization with *Sac*I and *Sal*I. The obtained novel constructs were called pET28a-AmSOD2.

The strain R-AmSOD2 was prepared by transforming the construct pET28a-AmSOD2 into Rosetta (DE3). A control strain which contained an empty pET28a (R-pET28a) was then applied as the control strain. The strain R-AmSOD1, which had been previously made by Fesharaki et al., was also used to produce AmSOD1 (a Cu/Zn SOD isoform) in this research^18^.

The mitochondrial transit peptide in the sequence of AmSOD2 was identified by TargetP-2.0 software^56^. The nucleotide sequence of AmSOD2 without transit peptide, which is known as tAmSOD2, was prepared by PCR. To do this, the primers were designed, and the PCR reaction was performed by employing Pfu DNA polymerase (Thermo Scientific) in a reaction mixture which contained template plasmid (pET28a-AmSOD2), deoxynucleotides, reaction buffer as well as the primers 5′ATATGAGCTCCTGCAGACTTTCTCGCT3′ which carried an *SacI* restriction site at the 5′end (underlined), and 5′ATATGTCGACTCACTTAATGC CCAATGAAT3′ with a *Hin*dIII restriction site (underlined) at the 3′ end. Four bases were also added and included at the 5′ end for each of the oligonucleotide primers. The thermal profile was as follows: 1 cycle at the temperature of 95 °C for a period of 5 min; 30 cycles at the temperature of 94 °C for a period of 1 min; 62 °C for a period 1 min; 72 °C for a period of 2 min and 1 cycle at the temperature of 72 °C for a period 10 min. Then the PCR product was digested with *Sac*I and *Sal*I and then ligated into pET-28a as the expression vector (Novagen) following linearization with *Sac*I and *Sal*I. The sequence of resulting plasmid, called pET28a-tAmSOD2, was verified by sequencing. Then the construct was introduced in the *E. coli* strain, Rosetta (DE3), for the expression of protein. The obtained strain was referred to as R-tAmSOD2.

### Heterologous expression of AmSOD2 and tAmSOD2 in *E. coli*

R-AmSOD2 and R-tAmSOD2 strains were grown at the temperature of 37 °C in Luria–Bertani (LB) medium with the 80 ml volume; it had been supplemented with 50 µg/ml kanamycin and 5 µg/mL chloramphenicol to an OD_600_ of 0.6. At this OD, inducing of the related cultures was done by 100 µM Isopropyl-d-1-thiogalactopyranoside (IPTG). For the confirmation of heterologous expression, 1.5 ml samples of each of the culture media were harvested through 1, 2, 3 and 4 h centrifugation following the IPTG addition and then frozen at the temperature of − 80 °C until being used. To extract soluble proteins, first, the cell pellets (as got from 1.5 ml cell culture) were allowed to be re-suspended in 750 µl buffers A (50 mM Tris–HCl, 50 mM Glucose, pH 7.8). The cells were then harvested by centrifuging at the temperature of 4 °C for a period of 20 min. The harvested cells were then again subjected to re-suspending in a 56 µl buffer A contained 1 mg/ml lysozyme. The cell suspensions were subsequently kept at room temperature for a period of 15 min. Then 56 µl of buffer B (50 mM Tris–HCl, 50 mM KCl, 1% Triton X-100) was added and the shaking of the cell suspensions was performed for a period of 2 h at the temperature of 37 °C. The centrifugation of the cell suspension was done (25 min, 4 °C, and 10000*g*) for the removal of cell debris. The supernatant which contained soluble protein was then transferred to clean tubes; the pellets were then kept at the temperature of − 80 °C. The concentration of total extracted soluble protein was then determined through Bradfod assay by employing BSA as standard^57^. To load the supernatant which contained the soluble fraction on the SDS-PAGE, addition of 4 µl of the loading buffer to 20 µl of soluble extract was done; then it was heated at the temperature of 95 °C for a period of 3 min. Loading of 20 µl of such a sample on the 12% SDS-PAGE was done, as described before, following a short spin^58^. Staining of SDS-PAGE was then done with Coomassi Brilliant Blue G250^59^.

To extract total protein (the total of soluble and insoluble proteins), the frozen pellets (as obtained from 2 ml cell culture) were suspended in 200 µL of buffer 1 (10 ml Tris–HCl pH 8). Then 40 µl of buffer 2 (Tris–HCl pH 6.8, 10% SDS, 50% Glycerol and 4 µl DTT) was added to the previous suspension. The heating of the solution was done at 95 °C. The harvesting of cell debris was done by centrifuging for a period of 5 min and 20 µl of the supernatant was loaded on SDS-PAGE^18^.

To compare the activity of AmSOD2 with AmSOD1, the production of recombinant form of AmSOD1 was also performed as described before^18^.

To analyze His-AmSOD2 and His-tAmSOD2 stability, the corresponding bands intensity in the total protein which was extracted from each of the strains was compared with that obtained in the soluble faction. Determination of the soluble extract's concentration was done by applying Bradford assay, through BSA as the standard^57^.

### Purification of His-AmSODs

The considered soluble proteins were then used for His-Trap HP columns (GE healthcare) and pre-equilibrated with the loading buffer (10 mM imidazole, 500 mM NaCl, 30 mM Tris–HCl, pH 8.0); then it was eluted in some 10–200 mM imidazole gradient^60,61^. After that, the removal of imidazole was done by dialysis against Tris–HCl pH 8. Owing to the aromatic amino acid’s absence in AmSOD1, AmSOD2 and tAmSOD2, the concentration of purified His-AmSOD1, His-AmSOD2 and His-tAmSOD2 was determined by A205 divided by 31 as the extinction coefficient.

### Determining enzyme's native mass

The determination of the solution molecular mass related to the native enzyme was done by applying gel filtration chromatography according to its elution time, based on the comparison with the standard proteins.

### Quantification of metal ions

To determine metal contents in AmSOD2, drying of the purified and desalted tAmSOD2 was done at the temperature of 110 °C and digestion was carried out with 0.5 ml concentrated nitric acid (HNO_3_) for a period of 2 h at the temperature of 95 °C^61^. Following cooling at the room temperature, dilution of the digested products was done in deionized water to reach 5 ml as the final volume and subjected to analysis Mn^2+^ by inductively coupled plasma atomic emission spectroscopy (ICP- AES, OPTIMA 7300DV).

### SOD activity

The strain R-AmSOD1, R-AmSOD2 and R-tAmSOD2, which were grown in the LB medium, as described above. To evaluate AmSOD2 and tAmSOD2 in the presence of Mn^2+^, the medium was supplemented with 1 mM MnCl_2_, respectively, 20 min after the addition of IPTG. Extraction of the soluble protein was done, as shown above. Determination of the SOD activity was performed for crude protein as well as purified SODs. In this method, production of the superoxide anion is done by oxidizing xanthine through xanthine oxidase^62^. Briefly, hypoxanthine and xanthine oxidase were used for the generation of free radicals; these, in turn, have a reaction with tetrazolium salt for the formation of a formazan dye which has absorbance at 440–460 nm. One SOD unit is that causing 50% inhibition in the reduction rate of tetrazolium under the assay conditions. The obtained unit was then divided by the soluble protein's amount (as determined by Bradford assay) or purified protein (specified by absorbance at 205 nm) and the activities were represented as U/mg.

To study the effect of inhibitors on the activity of His-tAmSOD2, the activity of this enzume was determined after treatment of enzymes with various concentrations of sodium azide, diethyldithiocarbamate, and potassium cyanide. Then the activity of protein was determined as explained above. The percentage of inhibition of activity by these inhibitors was calculated based on the activity of protein with no treatment.

### Stability of tAmSOD2 in diverse pH and temperatures

The enzyme’s stability at diverse temperatures was investigated following purification of His-tAmSOD2. The aliquots of the purified enzyme were incubated at diverse temperatures (25, 37, 50, 60, 75 °C) in 0.1 M potassium phosphate buffer, pH 8, for a period of 1 h. The enzyme’s aliquots were then kept on ice for the activity assay. To study pH, the enzyme aliquots were incubated at various pH levels (3, 4, 5, 6, 7, 8, 9, 10, 11, and 12) for a period of 1 h at room temperature. For pH 4–5; 0.1 M Glycine–HCl buffer, for pH 6–8; 0.1 M potassium phosphate buffer and for pH 9–10; 0.1 M Glycine–NaOH was used. The enzyme aliquots were then kept on ice for the activity assay. For temperature, the ratio of activities was calculated based on the activity of fresh pure recombinant His-tAmSOD (No treatment). For pH, the ratio was calculated based on the protein activity in pH 6. Each data point represents the mean ± SD obtained from three independent reactions.

### Longevity of His-tAmSOD2

Effect of long-term storage on the stability of His tAmSOD1 before and after dialysis was evaluated up to 3 months of storage at 25, 4 and 20 °C using the activity assay.

### Sequence analysis

Multiple alignments of selected plant SODs in addition with AmSOD1 were performed using ClustalW (http://www.ebi.ac.uk/Tools/clustalw). An unrooted phylogenetic tree was constructed based on alignment by a maximum-likelihood method using the MEGA 5.2 version^63^. The accession number introduced at the National Center for Biotechnology Information (NCBI) website for SOD protein family were as following: At, *Arabidopsis thaliana*, AtSOD1 (NP-172360.1), AtSOD2 (NP-565666.1), AtSOD3 (NP-001119245.1), AtSOD4 (AAC24832.1); JC, *Jatropha curcas*, JCSOD (AGW52120.1); Cr, *Capsella rubella,* CrSOD (XP_006294965.1); Oe, *Olea europaea*, OeSOD (XP_022849259.1); Am, *Avicennia marina*, AmSOD1 (ACA50531.1), AmSOD2 (AAN15216.1); Si, *Sesamum indicum,* SiSOD (NP_001306618.1); Hi, *Handroanthus impetiginosus*, HiSOD (PIN20638.1); Ae, *Acanthus ebracteatus*, AeSOD (ABK32075.1); Xs, *Xanthoceras sorbifolium*, XsSOD (AVD97170.1); Hv, *Hordeum vulgare*, HvSOD (KAE8811372.1), Os, *Oryza sativa*, OsSOD (BAA37131.1); Nn, *Nelumbo nucifera*, NnSOD (ABA10481.1); Sl, *Solanum lycopersicum*, SlSOD (AAQ18699.1); Ht, *Helianthus tuberosus*, HtSOD (QCD25908.1); Ad, *Actinidia deliciosa,* AdSOD (ASL04620.1), Xs, *Xanthoceras sorbifolium*, XsSOD (AVD97167.1).

### Statistical analysis

All data were collected and entered into an Excel spreadsheet .Statistical analysis was performed using IBM SPSS Statistics 29.0. The data were presented as mean ± standard deviation (SD) from three independent biological replications. The statistical significance of the difference was determined by LSD test. Differences between treatments were considered significant when p < 0.05.

### Supplementary Information

Supplementary Figure S1.

## Data Availability

The datasets used and analysed during the current study is available from the corresponding author on reasonable request.

## Abbreviations

AmSOD1: *Avicennia marina* CuZnSOD
AmSOD2: *Avicennia marina* MnSOD
IPTG: Isopropyl β-d-1-thiogalactopyranoside
ROS: Reactive oxygen apecies
