# Supplementary material for: Functional characterization of a manganese superoxide dismutase from Avicennia marina: insights into its role in salt, hydrogen peroxide, and heavy metal tolerance
Source: Sci Rep. 2024 Jan 3;14:406. doi: 10.1038/s41598-023-50851-5 (PMC10764323; doi:10.1038/s41598-023-50851-5)
Supplement: Supplementary file 1 — Supplementary Figure S1. [file 41598_2023_50851_MOESM1_ESM.docx]

**Supplementary Fig S1.-A- Multiple alignment between SOD isoforms in plants**

AmSOD2 ------------------MALRALVTRNPLRAPS--------LTCRGLQTFSLPDLPYDY

SiSOD ------------------MALRALVTRRTLRTSP--------IGFRGLQTFSLPDLTYDY

AeSOD -------------------ALRTLVTRKTLRAFP--------VGFRGLQTFSLPDLPYDY

HiSOD ------------------MALRTLVSRKTLRTS--------SLGLRGLQTFSLPDLPYDY

TanSOD ------------------MALRTLAAKKTLTTARQGLA---LIQSRSLQTFSLPDLSYDY

XsSOD2 ------------------MALRSLVTPGTLTRGAKS-V---GLGLRGLHTFTLPDLPYDY

NnSOD ------------------MALRSVISRKTLGSLGLG-----FSHARGLQTFTLPDLPYDY

PvSOD ------------------MALLSYVTRKTLTESLRLGL---KSHVRGLQTFTLPDLPYEY

HaSOD ------------------MALRTLATRKTLGAFSTFP-----QQLRGLQTFTLPDLAYDY

AtSOD4 ------------------MAIRCVASRKTLAGLKETSSR--LLRIRGIQTFTLPDLPYDY

HvSOD ------------------MALRTLATKRTLGLALGGAR-------RGVATFTLPDLPYDY

TaSOD ------------------MALRTLAAKKTLGLALGGARPLAAA--RGVATFTLPDLPYDF

ScSOD ------------------MALRTLASKKALYFALGGAARPLAASSRGVTTVTLPDLSYDF

ZmSOD2 ------------------MALRTLASKNALSFALGGAARPSAASARGVTTVALPDLSYDF

OsSOD2 ------------------MALRTLASR--KTLAAAALPLAAAAAARGVTTVALPDLPYDY

OsSOD3 --------MAAFASALRVLPSPPAAVPRRLRSREQRQGCRSRRYSKVVAYYALTTPPYKL

XsSOD3 ------------MGSCICNPLSASRHLLLTESNSPKLPHSKRRASTVVAYYGLKTPPYKL

SlSOD --MAATASANSLTSAFLPPQGFNGSSKSLQWRTQKKQFGRKAGSATITAKFDLIPPPYPM

AdSOD MAAAAATTAISLTRAFIPCQ--GGSTRSLPFRKKERQCMRKAGPCMITAKFELKPPPYPL

HtSOD -MASVTTIASHPTSAFISTPEFSRLSQSLHSSLRQRRCSRKSVCGTISASIDLKPPPYAL

NnSOD3 ------------------------------------------------------------

AtSOD2 --------------------MAATNTILAFSSPSRLLIP------PSSNPSTLRSSFRGV

CrSOD --------------------MAAANTILAFSSPSRLLIPSSSTPSPSSSSSTLRSSFRGV

ZmSOD1 --------------------MAAQSFLLAATAA----------------ARSPAVFAAPY

TaSOD1 --------------------MAAQSLLFAAAAP----------------LFQAPASARPF

JcSOD -----------MQAAAAVAAMAAHTILAASPSSHPLLYPFPN---PILSHSSPLHSSFHG

OeSOD ------------------------------------------------------------

AmSOD1 ------------------------------------------------------------

AtSOD1 ------------------------------------------------------------

AtSOD3 ------------------------------------------------------------

AmSOD2 GALEPAISGEIMQLHHQKHHQTYITNYNKALEQLDGAIAKGDASTVVK-----LQSAIKF

SiSOD GSLEPAISGEIMQLHHQKHHQTYITNYNKALEQLDGAIAKGDSSTVVK-----LQSAIKF

AeSOD GALEPAISAEIMQLHHQKHHQTYITNYNKALEQLDGAISKGDASTVVK-----LQSAIKF

HiSOD GALEPAISGEIMQLHHQKHHQTYVTNFNKAIEQLDGAASKGDASTIVK-----LQSAIKF

TanSOD GALEPAISGEIMQLHHQKHHQTYVTNYDKALEQLDGAMSKGDAPTVVK-----LQSAIKF

XsSOD2 GALEPAISGEIMQLHHQKHHQTYITNYNKALEQLDEAITEGDASTVVK-----LQSAIKF

NnSOD SALEPAISVEIMRLHHQKHHQTYITNYNKALEQLEEAMAKGDSSAVVK-----LQAAIKF

PvSOD GALEPAISSEIMQLHHQKHHQTYITNYNKALEQLDQAINKGDASAVVK-----LQSAIKF

HaSOD GALEPAISGDIMQLHHQKHHQTYITNYNKALEQLDDAIAKGDASTAVK-----LQSAIKF

AtSOD4 GALEPAISGEIMQIHHQKHHQAYVTNYNNALEQLDQAVNKGDASTVVK-----LQSAIKF

HvSOD GALEPAVSGEIMRLHHQKHHATYVANYNKALEQLDAAVGKGDASGVVH-----LQSAIKF

TaSOD GALEPAVSGEIMRLHHQKHHATYVANYNKALEQLDAAVSKGDASAVVH-----LQSAIKF

ScSOD GALEPAISGEIMRLHHQKHHATYVANYNKALEQLDAAVAKGDASAVVQ-----LQGAIKF

ZmSOD2 GALEPAISGEIMRLHHQKHHATYVGNYNKALEQLDAAVAKGDASAVVQ-----LQGAIKF

OsSOD2 GALEPAISGEIMRLHHQKHHATYVANYNKALEQLDAAVAKGDAPAIVH-----LQSAIKF

OsSOD3 DALEPYISKRTVELHWGKHQQDYVDSLNKQLATS-MFYGYTLEELIKEAYNNGNPLPEYN

XsSOD3 DALEPYMSKRTLEMHWGEHHRDYVECLNKDLDKSDLFYGYPMDELIKFTYNNGNPLPEFK

SlSOD DALEPHMSSRTFEFHWGKYHRAYVDNLNKQIDGT-ELDGKTLEDIILVTYNNGAPLPAFN

AdSOD NALEPLMSRDTLEYHWGKHHRTYVENLNKQIVGT-ELDGMALEDIIVATYNKGDLLPAFN

HtSOD NALEPHMSKETLEYHWGKHHRGYVNNLNKQIEGT-ELHEKSLESIILASYNKGDILPAFN

NnSOD3 --------------HWGKHHQTYVTNLNNLIKGT-DFEGKTLEEIVRSSDGG-----VFN

AtSOD2 SLNNNNLHRLQSVSFAVKAPSKALTVVSAAKKAVAVLKGTSDVEGVVT---------LTQ

CrSOD SLNN-LHRRPQSVSFSARAPSKSLTVVSAAKKAVAVLKGNSDVEGVVT---------LTQ

ZmSOD1 SSARPFHSVHFVAGPWGAAAAKALVVADATKKAVAVLKGASEVEGVVT---------LTQ

TaSOD1 QSLRIVSTPGGAT-----AAARALVVADATKKAVAVLKGSSQVEGVVT---------LTQ

JcSOD VSLKLPRQSLPLSLTTAAAPKKPLAVVAATKKAVAVLKGTSNVEGVVT---------LTQ

OeSOD -----------------------------MVKAVTVLNSSEGVTGTVY---------FTQ

AmSOD1 -----------------------------MPKAVAVLSSNEGVRGTVY---------FTQ

AtSOD1 -----------------------------MAKGVAVLNSSEGVTGTIF---------FTQ

AtSOD3 -----------------------MEAPRGNLRAVALIAGDNNVRGCLQ---------FVQ

AmSOD2 NGGGHVNHSIFWKNLAPVREGGGEPPKGSLGWAIDHDFGSLEALIQNMNAEGAALQGSGW

SiSOD NGGGHVNHSIFWKNLAPIREGGGEPPKGSLGWAIDNHFGSLDALIQKMNAEGAALQGSGW

AeSOD NGGGHVNHSIFWKNLAPVPEGGGEPPKGSLGSAVDNHFGSLDALIQKMNAQGAALQGSGW

HiSOD NGGGHVNHSIFWKNLAPVREGGGEPPKGSLGWAIDNHFGSLDALVQKINAEGAALQGSGW

TanSOD NGGGHINHSIFWKNLAPVYQGGGEPPKGNLGWRIDEDFGSLETLVQKMNAEGAALQGSGW

XsSOD2 NGGGHVNHSIFWKNLAPANEGGGEPPKGSLGWAIDNHFGSLEALIQKMNAEGAALQGSGW

NnSOD NGGGHINHSIFWKNLIPTSEGGGEPPHGALGWAIDTHFGSFEALVKKVNAEGAALQGSGW

PvSOD NGGGHINHSIFWKNLTPVSEGGGEPPHGSLGWAIDTNFGSMEALIQRMNAEGAALQGSGW

HaSOD NGGGHVNHSIFWKNLAPTKEGGGEPPHGSLGWAIDQSYGSVEKLIAKMNAEGAAVQGSGW

AtSOD4 NGGGHVNHSIFWKNLAPSSEGGGEPPKGSLGSAIDAHFGSLEGLVKKMSAEGAAVQGSGW

HvSOD NGGGHVNHSIFWKNLKPISEGGGEPPHGKLGWAIDEDFGSIEKLIKKMNAEGAALQGSGW

TaSOD NGGGHVNHSIFWKNLKPISEGGGEPPHGKLGWAIDEDFGSIEKLIKKMNAEGAALQGSGW

ScSOD NGGGHVNHSIFWKNLKPISEGGGEPPHGKLGWAIDEDFGSFEALVKKMNAEGAALQGSGW

ZmSOD2 NGGGHVNHSIFWKNLKPISEGGGEPPHGKLGWAIDEDFGSFEALVKRMNAEGAALQGSGW

OsSOD2 NGGGHVNHSIFWNNLKPISEGGGDPPHAKLGWAIDEDFGSFEALVKKMSAEGAALQGSGW

OsSOD3 NAAQVWNHHFFWESMQP---EGGGSPGRGVLQQIEKDFGSFTNFREEFIRSALSLLGSGW

XsSOD3 NAAQVWNHDFFWESMQP---GGGDMPKFGVLEQIEKDFGSFTNFREKFIEAALTLFGSGW

SlSOD NAAQAWNHQFFWESMKP---NGGGEPSGELLELINRDFGSYDTFVKEFKAAAATQFGSGW

AdSOD NAAQAWNHEFFWESMKP---GGGGEPSGELLKLVIRDFGSFEGFIKEFKAAAATQFGSGW

HtSOD NAAQVWNHEFFWESMKP---GGGGKPSRELLELIIRDFGSFEALIQEFKLAAATHFGSGW

NnSOD3 NAAQVWNHTFYWHCLAP---NAGGEPTGELAAAINAAFGSFADFKTKFTDAAVKNFGSGW

AtSOD2 DDSGPTTVNVRITGLTPGPHGFHLHEFGDTTNGCISTGPHFNPNNMTHGAPEDECRHAGD

CrSOD DDSGPTTVNVRITGLTPGPHGFHLHEFGDTTNGCISTGPHFNPNNMTHGAPEDECRHAGD

ZmSOD1 DDDGPTTVNVRITGLTPGLHGFHLHEFGDTTNGCISTGPHFNPNNLTHGAPEDEVRHAGD

TaSOD1 EDDGPTTVNVRITGLAPGLHGFHLHEFGDTTNGCISTGPHFNPNGLTHGAPEDEVRHAGD

JcSOD EDDGPTTVNVRVTGLTPGPHGFHLHEYGDTTNGCISTGAHFNPNNKTHGAPEDEIRHAGD

OeSOD EGDGPTTVTGNLSGLKPGLHGFHVHALGDTTNGCMSTGPHFNPVGKEHGAPGDENRHAGD

AmSOD1 EGDGPTTVTGNLSGFKSGPHGFHVHALGDTTNGCMSTGPHFNPAGKDHGAPEDEVRHAGD

AtSOD1 EGDGVTTVSGTVSGLKPGLHGFHVHALGDTTNGCMSTGPHFNPDGKTHGAPEDANRHAGD

AtSOD3 DISGTTHVTGKISGLSPGFHGFHIHSFGDTTNGCISTGPHFNPLNRVHGPPNEEERHAGD

: : . :*

AmSOD2 VWLAVDK----------------EFKRLVVETTANQDPLVTKGPSLVPLLGIDVWEHAYY

SiSOD VWLGVDK----------------ELKHLVVETTANQDPLVTKGPGLVPLLGIDVWEHAYY

AeSOD VWLGLDK----------------ESKHLVVETTANQDPLVTKGPSLVPLLGIDVWEHAYY

HiSOD VWLGVDK----------------ELKHLVVETTANQDPLVTKGPNLVPLLGIDVWEHAYY

TanSOD VWLGVDK----------------ESKKLVIETTANQDPLMTKGPNLVPLLGIDVWEHAYY

XsSOD2 VWLGLDK----------------EFKKLVVKTTANQDPLVTKGPSLVPLLGIDVWEHAYY

NnSOD VWLGVDK----------------ESQKLVVETTANQDPLVTKGPNLVPLLGIDVWEHAYY

PvSOD VWLGLDK----------------ESKKLVVETTANQDPLVTKGPSLVPLLGIDVWEHAYY

HaSOD VWLAVDK----------------ELKRLVVETTANQDPLVTKGPSLVPLIGIDVWEHAYY

AtSOD4 VWLGLDK----------------ELKKLVFDTTANQDPLVTKGGSLVPLVGIDVWEHAYY

HvSOD VWLALDK----------------EAKKLSVETTPNQDPLVTKGANLYPLLGIDVWEHAYY

TaSOD VWLALDK----------------EAKKLSVETTPNQDPLVTKGSNLYPLLGIDVWEHAYY

ScSOD VWLALDK----------------EAKKLSVETTANQDPLVTKGASLVPLLGIDVWEHAYY

ZmSOD2 VWLALDK----------------EPKKLSVETTANQDPLVTKGASLVPLLGIDVWEHAYY

OsSOD2 VWLALDK----------------EAKKLSVETTANQDPLVTKGANLVPLLGIDVWEHAYY

OsSOD3 VLLVLKR---------------KERKLSVVHTQNAISPLALG---DIPLINLDLWEHAYY

XsSOD3 VWLVLKR---------------EERRLEVIKTSNAISPLVWD---DIPIISLDMWEHAYY

SlSOD AWLAYKP---------------EDKKLALVKTPNAENPLVLG---YTPLLTIDVWEHAYY

AdSOD AWLAYKANRLDVGNAVNPLPSEEDKKLVVVKSPNAVNPLVWD---YSPLLTIDVWEHAYY

HtSOD TWLVCKC---------------------CVCSTKTHDQTI--------------------

NnSOD3 TWLVKEADG----------------KLAIVSTSNAGTPLTTS---ATPLMTVDVWEHAYY

AtSOD2 LGNINAN----------------ADGVAETTIVDNQIPLTGP----NSVVGRAFVVHELK

CrSOD LGNINAN----------------ADGVAETTIVDNQIPLTGP----NSVVGRAFVVHELK

ZmSOD1 LGNIVAN----------------AEGIAEATIVDTQIPLTGP----NSVVGRAFVVHELE

TaSOD1 LGNIVAN----------------AEGVAETTIVDSQIPLTGP----NAVVGRAFVVHELE

JcSOD LGNIVAN----------------ADGVAEATIVDNQIPLSGP----NAVVGRALVVHELE

OeSOD LGNITVG----------------EDGTAAINIVDKQIPLTGP----HSIIGRAVVVHSDP

AmSOD1 LGNITVG----------------EDGTAAVNIVDKQIPLSGP----HSIVGRAVVVHADP

AtSOD1 LGNITVG----------------DDGTATFTITDCQIPLTGP----NSIVGRAVVVHADP

AtSOD3 LGNILAG----------------SNGVAEILIKDKHIPLSGQ----YSILGRAVVVHADP

AmSOD2 LQYKNVRPDYLKNIWKVINWKYASEVYDSLGIK---------------------------

SiSOD LQYKNVRPDYLKNIWKVMNWKYASDVYDKECP----------------------------

AeSOD LQYKNVRPDYLKNIWKVINWKYAGEVYENESC----------------------------

HiSOD LQYKNVRPDYLKNIWKVINWKYAGEVYEKECP----------------------------

TanSOD LQYKNVRPDYLKNVWKVMHWKYAGEVYDKECPQL--------------------------

XsSOD2 LQYKNVRPDYMKNIWKVMNWKYASEVYDKENP----------------------------

NnSOD LQYKNVRPDYLNNIWKVINWKYAGEVYDKECPHPESR-----------------------

PvSOD LQYKNVRPDYLKNIWKVINWKYAGELYQKECP----------------------------

HaSOD LQYKNVRPDYLKNIWKVINWKYASEIYEKECP----------------------------

AtSOD4 LQYKNVRPEYLKNVWKVINWKYASEVYEKENN----------------------------

HvSOD LQYKNVRPDYLTNIWKVVNWKYAGEEYEKVLA----------------------------

TaSOD LQYKNVRPDYLTNIWKVVNWKYAGEEYEKVLA----------------------------

ScSOD LQYKNVRPDYLNNIWKVINWKYAGEVYENVLA----------------------------

ZmSOD2 LQYKNVRPDYLNNIWKVMNWKYAGEVYENVLA----------------------------

OsSOD2 LQYKNVRPDYLSNIWKVMNWKYAGEVYENATA----------------------------

OsSOD3 LDYKDDRRMYVTNFIDHLVSWDTVTLRMMRAEAFVNLGEPNIPVA---------------

XsSOD3 LDYKNDKAKYVNVFMNHLVSWNAAMSRMARAEAFVNLGEPKIPVA---------------

SlSOD LDFQNRRPDYISIFMEKLVSWEAVSIRLKAASA---------------------------

AdSOD LDFQNRRPDYISTFMEKLVSWEAVSLRLEKAKARATEREKEEERRREQEEGKMADDETTE

HtSOD ------------------------------------------------------------

NnSOD3 ------------------------------------------------------------

AtSOD2 DDLGKGGHELSLTTGNAGGRLACGVIGLTPL-----------------------------

CrSOD DDLGKGGHELSLTTGNAGGRLACGVIGLTPL-----------------------------

ZmSOD1 DDLGKGGHELSLSTGNAGGRLACGVVGLTPL-----------------------------

TaSOD1 DDLGKGGHELSLSTGNAGGRLACGVVGLTPL-----------------------------

JcSOD DDLGKGGHELSLTTGNAGGRLACGLLAMCKAAWLSQCKEMGSEVDT--------------

OeSOD DDLGRGGHELSKSTGNAGGRVACGIIGLQG------------------------------

AmSOD1 DDLGKGGHELSKTTGNAGGRVACGIVGLQG------------------------------

AtSOD1 DDLGKGGHELSLATGNAGGRVACGIIGLQG------------------------------

AtSOD3 DDLGKGTKH---------------------------------------------------

AmSOD2 -------------

SiSOD -------------

AeSOD -------------

HiSOD -------------

TanSOD -------------

XsSOD2 -------------

NnSOD -------------

PvSOD -------------

HaSOD -------------

AtSOD4 -------------

HvSOD -------------

TaSOD -------------

ScSOD -------------

ZmSOD2 -------------

OsSOD2 -------------

OsSOD3 -------------

XsSOD3 -------------

SlSOD -------------

AdSOD THLESDADDSEAK

HtSOD -------------

NnSOD3 -------------

AtSOD2 -------------

CrSOD -------------

ZmSOD1 -------------

TaSOD1 -------------

JcSOD -------------

OeSOD -------------

AmSOD1 -------------

AtSOD1 -------------

AtSOD3 -------------

**Fig. S1-B- Multiple alignment betweem MnSOD amino acid sequences from different plants**

**Blue Box:Unconserved Sequences Red Boxes:Conserved Sequences**

HiSOD MALRTLVSRKTLR-TS-----SLGLR--GLQTFSLPDLPYDYGALEPAISGEIMQLHHQK

TanSOD MALRTLAAKKTLT-TARQGLALIQSR--SLQTFSLPDLSYDYGALEPAISGEIMQLHHQK

AmSOD2 MALRALVTR------NPLRAPSLTCR--GLQTFSLPDLPYDYGALEPAISGEIMQLHHQK

SiSOD MALRALVTR------RTLRTSPIGFR--GLQTFSLPDLTYDYGSLEPAISGEIMQLHHQK

AeSOD -ALRTLVTR------KTLRAFPVGFR--GLQTFSLPDLPYDYGALEPAISAEIMQLHHQK

XsSOD MALRSLVTPG-TL-TRGAKSVGLGLR--GLHTFTLPDLPYDYGALEPAISGEIMQLHHQK

NnSOD MALRSVISRKTLG-SLGLG--FSHAR--GLQTFTLPDLPYDYSALEPAISVEIMRLHHQK

PvSOD MALLSYVTRKTLT-ESLRLGLKSHVR--GLQTFTLPDLPYEYGALEPAISSEIMQLHHQK

HaSOD MALRTLATRKTLG---AFSTFPQQLR--GLQTFTLPDLAYDYGALEPAISGDIMQLHHQK

AtSOD4 MAIRCVASRKTLAGLKETSSRLLRIR--GIQTFTLPDLPYDYGALEPAISGEIMQIHHQK

HvSOD MALRTLATKRTLGLALGGAR-------RGVATFTLPDLPYDYGALEPAVSGEIMRLHHQK

TaSOD MALRTLAAKKTLGLALGGARPLAAA--RGVATFTLPDLPYDFGALEPAVSGEIMRLHHQK

ScSOD MALRTLASKKALYFALGGAARPLAASSRGVTTVTLPDLSYDFGALEPAISGEIMRLHHQK

ZmSOD MALRTLASKNALSFALGGAARPSAASARGVTTVALPDLSYDFGALEPAISGEIMRLHHQK

OsSOD MALRTLASR--KTLAAAALPLAAAAAARGVTTVALPDLPYDYGALEPAISGEIMRLHHQK

*: : .: *.:****.*::.:****:* :**::****

HiSOD HHQTYVTNFNKAIEQLDGAASKGDASTIVKLQSAIKFNGGGHVNHSIFWKNLAPVREGGG

TanSOD HHQTYVTNYDKALEQLDGAMSKGDAPTVVKLQSAIKFNGGGHINHSIFWKNLAPVYQGGG

AmSOD2 HHQTYITNYNKALEQLDGAIAKGDASTVVKLQSAIKFNGGGHVNHSIFWKNLAPVREGGG

SiSOD HHQTYITNYNKALEQLDGAIAKGDSSTVVKLQSAIKFNGGGHVNHSIFWKNLAPIREGGG

AeSOD HHQTYITNYNKALEQLDGAISKGDASTVVKLQSAIKFNGGGHVNHSIFWKNLAPVPEGGG

XsSOD HHQTYITNYNKALEQLDEAITEGDASTVVKLQSAIKFNGGGHVNHSIFWKNLAPANEGGG

NnSOD HHQTYITNYNKALEQLEEAMAKGDSSAVVKLQAAIKFNGGGHINHSIFWKNLIPTSEGGG

PvSOD HHQTYITNYNKALEQLDQAINKGDASAVVKLQSAIKFNGGGHINHSIFWKNLTPVSEGGG

HaSOD HHQTYITNYNKALEQLDDAIAKGDASTAVKLQSAIKFNGGGHVNHSIFWKNLAPTKEGGG

AtSOD4 HHQAYVTNYNNALEQLDQAVNKGDASTVVKLQSAIKFNGGGHVNHSIFWKNLAPSSEGGG

HvSOD HHATYVANYNKALEQLDAAVGKGDASGVVHLQSAIKFNGGGHVNHSIFWKNLKPISEGGG

TaSOD HHATYVANYNKALEQLDAAVSKGDASAVVHLQSAIKFNGGGHVNHSIFWKNLKPISEGGG

ScSOD HHATYVANYNKALEQLDAAVAKGDASAVVQLQGAIKFNGGGHVNHSIFWKNLKPISEGGG

Zm HHATYVGNYNKALEQLDAAVAKGDASAVVQLQGAIKFNGGGHVNHSIFWKNLKPISEGGG

OsSOD HHATYVANYNKALEQLDAAVAKGDAPAIVHLQSAIKFNGGGHVNHSIFWNNLKPISEGGG

** :*: *:::*:***: * :**:. *:**.*********:******:** * :***

HiSOD EPPKGSLGWAIDNHFGSLDALVQKINAEGAALQGSGWVWLGVDKELKHLVVETTANQDPL

TanSOD EPPKGNLGWRIDEDFGSLETLVQKMNAEGAALQGSGWVWLGVDKESKKLVIETTANQDPL

AmSOD2 EPPKGSLGWAIDHDFGSLEALIQNMNAEGAALQGSGWVWLAVDKEFKRLVVETTANQDPL

SiSOD EPPKGSLGWAIDNHFGSLDALIQKMNAEGAALQGSGWVWLGVDKELKHLVVETTANQDPL

AeSOD EPPKGSLGSAVDNHFGSLDALIQKMNAQGAALQGSGWVWLGLDKESKHLVVETTANQDPL

XsSOD EPPKGSLGWAIDNHFGSLEALIQKMNAEGAALQGSGWVWLGLDKEFKKLVVKTTANQDPL

NnSOD EPPHGALGWAIDTHFGSFEALVKKVNAEGAALQGSGWVWLGVDKESQKLVVETTANQDPL

PvSOD EPPHGSLGWAIDTNFGSMEALIQRMNAEGAALQGSGWVWLGLDKESKKLVVETTANQDPL

HaSOD EPPHGSLGWAIDQSYGSVEKLIAKMNAEGAAVQGSGWVWLAVDKELKRLVVETTANQDPL

AtSOD4 EPPKGSLGSAIDAHFGSLEGLVKKMSAEGAAVQGSGWVWLGLDKELKKLVFDTTANQDPL

HvSOD EPPHGKLGWAIDEDFGSIEKLIKKMNAEGAALQGSGWVWLALDKEAKKLSVETTPNQDPL

TaSOD EPPHGKLGWAIDEDFGSIEKLIKKMNAEGAALQGSGWVWLALDKEAKKLSVETTPNQDPL

ScSOD EPPHGKLGWAIDEDFGSFEALVKKMNAEGAALQGSGWVWLALDKEAKKLSVETTANQDPL

Zm EPPHGKLGWAIDEDFGSFEALVKRMNAEGAALQGSGWVWLALDKEPKKLSVETTANQDPL

OsSOD DPPHAKLGWAIDEDFGSFEALVKKMSAEGAALQGSGWVWLALDKEAKKLSVETTANQDPL

:**:. ** :* :**.: *: .:.*:***:********.:*** ::* ..**.*****

HiSOD VTKGPNLVPLLGIDVWEHAYYLQYKNVRPDYLKNIWKVINWKYAGEVYEKECP-----

TanSOD MTKGPNLVPLLGIDVWEHAYYLQYKNVRPDYLKNVWKVMHWKYAGEVYDKECPQL---

AmSOD2 VTKGPSLVPLLGIDVWEHAYYLQYKNVRPDYLKNIWKVINWKYASEVYDSLGIK----

SiSOD VTKGPGLVPLLGIDVWEHAYYLQYKNVRPDYLKNIWKVMNWKYASDVYDKECP-----

AeSOD VTKGPSLVPLLGIDVWEHAYYLQYKNVRPDYLKNIWKVINWKYAGEVYENESC-----

XsSOD VTKGPSLVPLLGIDVWEHAYYLQYKNVRPDYMKNIWKVMNWKYASEVYDKENP-----

NnSOD VTKGPNLVPLLGIDVWEHAYYLQYKNVRPDYLNNIWKVINWKYAGEVYDKECPHPESR

PvSOD VTKGPSLVPLLGIDVWEHAYYLQYKNVRPDYLKNIWKVINWKYAGELYQKECP-----

HaSOD VTKGPSLVPLIGIDVWEHAYYLQYKNVRPDYLKNIWKVINWKYASEIYEKECP-----

AtSOD4 VTKGGSLVPLVGIDVWEHAYYLQYKNVRPEYLKNVWKVINWKYASEVYEKENN-----

HvSOD VTKGANLYPLLGIDVWEHAYYLQYKNVRPDYLTNIWKVVNWKYAGEEYEKVLA-----

TaSOD VTKGSNLYPLLGIDVWEHAYYLQYKNVRPDYLTNIWKVVNWKYAGEEYEKVLA-----

ScSOD VTKGASLVPLLGIDVWEHAYYLQYKNVRPDYLNNIWKVINWKYAGEVYENVLA-----

Zm VTKGASLVPLLGIDVWEHAYYLQYKNVRPDYLNNIWKVMNWKYAGEVYENVLA-----

OsSOD VTKGANLVPLLGIDVWEHAYYLQYKNVRPDYLSNIWKVMNWKYAGEVYENATA-----

:*** .* **:******************:*:.*:***::****.: *:.
